# A Unique Case of Facial Hypervascularity Responding to Pulsed-Dye Laser

**DOI:** 10.7759/cureus.28726

**Published:** 2022-09-03

**Authors:** George Shaker, Ashraf Shaker

**Affiliations:** 1 Dermatology, Nova Southeastern University Dr. Kiran C. Patel College of Osteopathic Medicine, Fort Lauderdale, USA; 2 Emergency Medicine, Hackensack University Medical Center, Hackensack, USA

**Keywords:** rosacea, aberrant vessel, pulsed dye laser, facial erythema, hypervascularity

## Abstract

Facial hypervascularity is a condition that manifests as erythema and edema caused by aberrant blood vessels. Often, the cause of these abnormal blood vessels can be attributed to previous trauma or vascular conditions such as rosacea, although sometimes the cause is unknown. Pulsed dye laser (PDL) can be an effective treatment even when the cause is unknown.

We present a case of a 24-year-old male presenting with intermittent swelling, redness, and throbbing sensations of the nose and cheeks for the past five years. Physical examination was notable for prominent erythema and swelling of the nasal skin and mild erythema on the cheeks. He underwent treatment with PDL and achieved complete resolution of his symptoms. This case illustrates the effectiveness of PDL in the treatment of facial hypervascularity.

## Introduction

Facial hypervascularity is caused by the formation of aberrant blood vessels usually stemming from previous trauma, such as surgery. Damage to the tissue leads to the release of mediators that promote neovascularization during the healing process [1]. When the healing process does not trend normally, aberrant neovascularization may occur and lead to erythema, swelling, and discomfort. Facial hypervascularity may also be seen in patients with rosacea, an inflammatory skin condition characterized by erythema, papules, pustules, and telangiectasias, usually affecting the face. Rosacea is defined by persistent reddening of the central portion of the face lasting three months or more [2]. It is a chronic condition affecting more than 16 million Americans, with a disproportionate number of fair-skinned individuals between the ages of 30 and 60 years being affected [3]. In some cases of facial hypervascularity, the cause is unknown. Regardless of the etiology, pulsed dye laser (PDL) is a non-invasive approach to obliterating abnormal blood vessels and, consequently, eliminating the associated erythema and edema.

## Case presentation

We present a case of a 24-year-old male with no significant past medical history presenting with intermittent swelling, redness, and throbbing sensation of the nose and cheeks for the past five years. The episodes are triggered by heat, eating heavy meals, and strong emotions and last anywhere from minutes to hours at a time. He also notes that the erythema and swelling can be rapidly reversed by immersing the skin in a cold body of water or by chewing ice cubes. He has failed treatment with metronidazole topical gel 1%, azelaic acid foam 15%, oxymetazoline hydrochloride 1% cream, ivermectin 1% cream, low dose Accutane (20 mg/day), and intense pulsed light (IPL) targeted laser therapy. He also states that at the age of 23 years, he visited an otolaryngologist who ablated the posterior nasal nerve using cryotherapy, believing that to be the cause of symptoms, but no improvement was noted. Physical examination was notable for prominent erythema of the nasal skin and mild erythema on the cheeks. No other skin abnormalities were noted.

**Figure 1:**
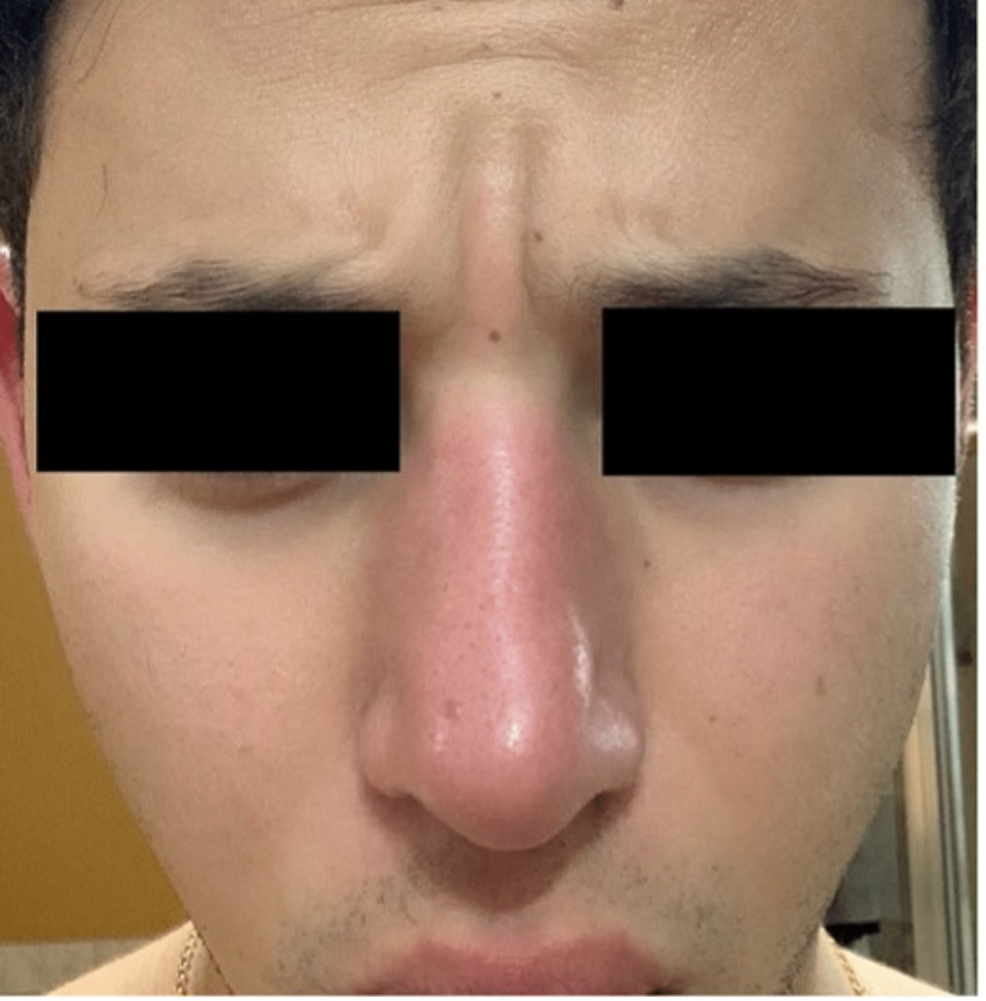
Prominent nasal erythema and swelling with mild erythema of the bilateral cheeks.

**Figure 2:**
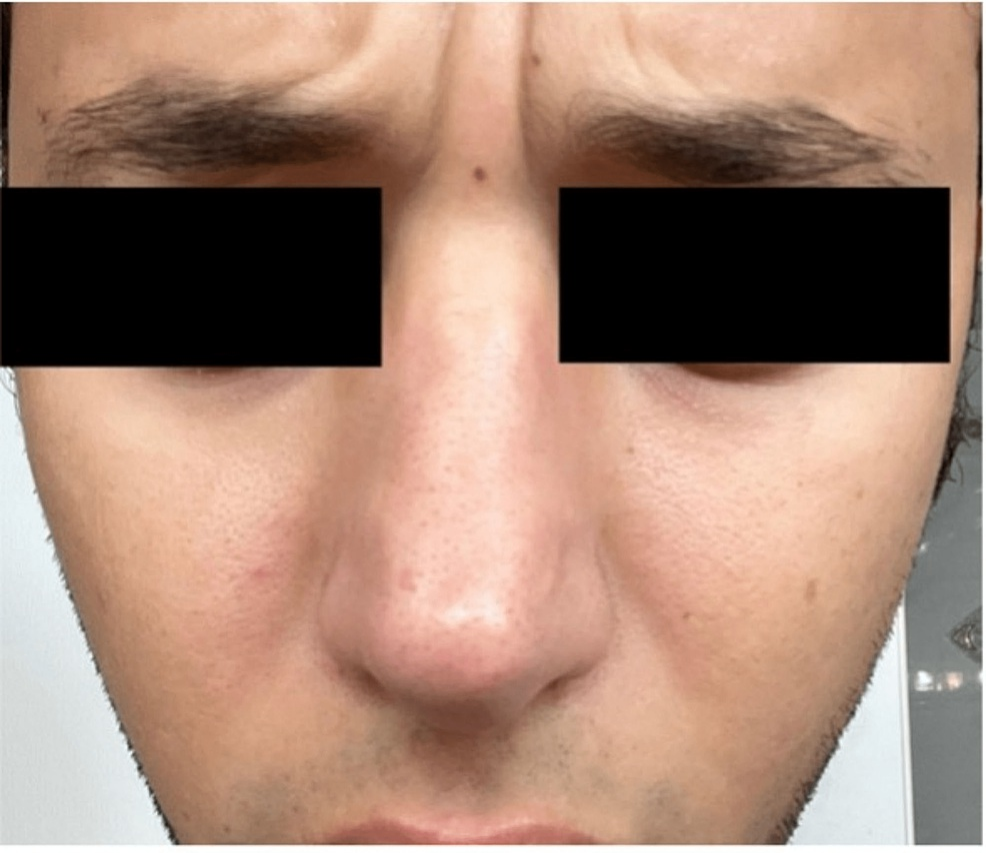
Significant reduction of nasal erythema and swelling.

Based on history and physical examination, a diagnosis of facial hypervascularity was reached. The patient underwent treatment with pulsed dye laser that was set at 595 nm wavelength, 6 ms pulse duration, and a fluence of 10 J/cm^2^. A total of 51 pulses were delivered. A photograph was taken before the first PDL treatment (Figure 1). The patient was advised to return in one month to repeat treatment. After completing three total sessions, each separated by four weeks, the patient achieved significant results. At the six-month follow-up appointment, he states that he has not had another episode of his symptoms (Figure 2).

## Discussion

PDL works based on the principle of selective thermolysis, which uses light to heat and destroy a target structure. In the case of cutaneous hypervascularity, the targeted structure is oxyhemoglobin which is contained within blood vessels [4]. PDL produces pulses of visible light at a wavelength of 585 or 595 nm. At this particular wavelength range, the target chromophore oxyhemoglobin is effectively targeted, which leads to higher absorption of the light energy by oxyhemoglobin and less scatter of the energy. The light energy is converted to heat which damages the blood vessels while leaving the surrounding skin undamaged. The damaged vessels are then resorbed by the body.

Other settings on PDL machines include power density (J/cm^2^) and pulse duration. Power density is a measurement of how much energy is delivered per area. As the power density is increased, the magnitude of the laser interaction with the target tissue is increased, and the damage to blood vessels is more pronounced [5]. The typical power density range is, on average, between 5 and 10 J/cm^2^. Pulse duration is the time it takes for the laser energy to reach the target tissue. The typical range for pulse duration is 0.45 to 40 milliseconds, with decreasing values indicating less time for the energy to reach the target tissue. Once the target absorbs the energy, the heat produced damages the target blood vessels, and the body’s natural healing process begins to resorb the damaged blood vessels, resulting in a reduction of symptoms [6].

Treatment with pulsed dye laser is typically painless, but some patients may describe a sensation similar to that of the “snap of a rubber band” [7]. Some patients may have bruising, skin irritation, and worsening erythema post-treatment, but these side effects typically resolve within three days of treatment. After treatment, it is advised to use a skin moisturizer to help protect the skin as well as sunscreen to reduce the chance of developing skin pigmentation.

Facial hypervascularity is a clinical diagnosis made when signs and symptoms are visibly apparent. In this case, neovascularization due to trauma or prior surgeries was excluded due to the patient’s lack of previous surgeries or trauma to the area. Rosacea was also considered but was unlikely due to failed outpatient treatment with all known medications for the condition. In addition, the patient did not fit the typical demographic and his rapid fluctuations of symptoms were inconsistent with the rosacea disease pattern. The patient was diagnosed with idiopathic facial hypervascularity and was successfully treated with PDL. To the best of our knowledge, this is the first report describing idiopathic facial hypervascularity and its successful treatment with PDL.

## Conclusions

PDL has a significant role in the treatment of vascular conditions. It works by emitting light energy at a specific wavelength that targets oxyhemoglobin. Because of its precision, it causes minimal to no damage to surrounding structures and eradicates only the targeted vessels. The injured vessels are eventually resorbed by the body which leads to a reduction in symptoms associated with the overgrowth or abnormal vasodilation of cutaneous vessels. PDL is a reasonable treatment for vascular conditions as it is minimally invasive and is highly effective for vascular conditions presenting with erythema or swelling.
